# Src Inhibition Can Synergize with Gemcitabine and Reverse Resistance in Triple Negative Breast Cancer Cells via the AKT/c-Jun Pathway

**DOI:** 10.1371/journal.pone.0169230

**Published:** 2016-12-30

**Authors:** Zhen-Hua Wu, Chen Lin, Ming-Ming Liu, Jian Zhang, Zhong-Hua Tao, Xi-Chun Hu

**Affiliations:** 1 Department of Medical Oncology, Fudan University Shanghai Cancer Center; Department of Oncology, Shanghai Medical College, Fudan University, Shanghai, China; 2 Department of Medical Chemistry, School of Pharmacy, Fudan University, Shanghai, China; University of South Alabama, UNITED STATES

## Abstract

**Purpose:**

Gemcitabine-based chemotherapy remains one of the standards in management of metastatic breast cancer. However, intrinsic and acquired resistance to gemcitabine inevitably occurs. The aims of this study were to assess the efficacy of the combination of src inhibition and gemcitabine in gemcitabine-resistant breast cancer cells.

**Methods and Results:**

By using colony formation, sphere forming, flow cytometry, cell counting kit-8 and transwell assays, 231/GEM-res (gemcitabine-resistant) cell line, which was 10 times more resistant, was shown to have elevated drug tolerance, enhanced proliferative and self-renewal abilities, compared with its parental cells. Inhibition of src by both saracatinib (AZD0530) and siRNA could partially reverse gemcitabine resistance and attenuate resistance-associated anti-apoptosis, migration and stem cell capacities. In addition, the combination of src inhibition and gemcitabine had synergistic antitumor effects. Western blot analysis revealed up-regulation of pro-apoptotic protein BAX, along with the down-regulation of anti-apoptotic proteins (BCL-XL, Survivin), migration associated proteins (p-FAK, MMP-3) and cancer stem cell (CSC) markers (CD44, Oct-4), which was probably mediated by AKT/c-Jun pathway.

**Conclusion:**

In highly gemcitabine-resistant 231 cells, src inhibition can synergize with gemcitabine, reverse drug resistance, inhibit tumor growth/metastasis/stemness of cancer stem cells, possibly via the AKT/c-Jun pathway.

## Introduction

Triple-negative breast cancer (TNBC) accounts for approximately 15% of breast cancers, which is associated with aggressive behavior, high risk of recurrence and worse prognosis [1, 2]. The lack of validated molecular targets, such as estrogen receptor (ER), progesterone receptor (PR), and human epidermal growth factor receptor-2 (HER-2), makes TNBC treatment particularly challenging [3]. Cytotoxic chemotherapy is currently the major therapeutic option and gemcitabine-based regimens have demonstrated extensive activity against advanced TNBC [4]. Unfortunately, chemo-resistance to gemcitabine is almost inevitable for these patients, and the underlying molecular mechanisms remain obscure.

src, a membrane-associated non-receptor tyrosine kinase, is the protein product of the proto-oncogene c-src. It participates in the activation of various downstream pathways involved in cell survival, angiogenesis, proliferation and motility [5]. Aberrant activation or overexpression of src and src-family kinases (SFK) has been observed in various tumors, including breast cancer, which is associated with metastatic progression and poor outcome [6, 7]. Here, MDA-MB-231, a ER/PR/Her-2 negative cell line and its gemcitabine resistant subline (231/GEM) were used. src kinase activity was significantly elevated in gemcitabine-resistant breast cancer cells. We hypothesized that src inhibition may help to overcome gemcitabine resistance, and then assessed the effects of different src expression status on development and reversal of chemo-resistance of TNBC.

In the study, we investigated the synergistic effect of src inhibition with gemcitabine in inhibition of multiple aspects of the malignant phenotype of gemcitabine resistant breast cancer cells, and provided insight into the possible mechanisms involved. Our findings indicate that the combination of src inhibition and gemcitabine may be a potential therapeutic strategy to sensitize gemcitabine-resistant breast cancer cells to gemcitabine through AKT/c-Jun pathway.

## Materials and Methods

### Cell lines and cell culture

The human breast cancer cell line MDA-MB-231 (231) was obtained from American Type Culture Collection (ATCC). MDA-MB-231 gemcitabine-resistant cells (231/GEM) were generously gifted by Xiaoli Yang from Key Laboratory of Breast Cancer in Fudan University Shanghai Cancer Center and they were generated by exposure to gradually increased concentrations of gemcitabine for more than one year [8]. Cells were cultured in DMEM supplemented with 10% fetal bovine serum (FBS) at 37°C in a humidified atmosphere with 5% CO_2_. 231/GEM cancer stem cells were enriched by serum-free suspending culture method involving supplements (DMEM-F12 with basic fibroblast growth factor: 10ng/mL, epidermal growth factor: 20ng/mL, bovine serum albumin: 0.4%, 50×B27: 4ml/L) under ultralow attachment condition.

### Drugs and reagents

Saracatinib(AZD0530) and PI3K inhibitor Duvelisib (IPI-145, INK1197) were purchased from Selleck Chemical (Houston, TX, USA). Gemcitabine was purchased from Lilly France (St-Cloud, France). Antibodies against β-actin (1:2000), CD44 (1:1000), Oct-4 (1:1000), SRC (1:1000), p-SRC (Tyr416) (1:1000), BCL-XL (1:1000), Survivin (1:1000), BAX (1:1000), FAK (1:1000), p-FAK (Tyr397) (1:1000), c-Jun (1:1000), p-c-Jun (Ser63) (1:1000), AKT (1:1000), p-AKT (Ser473) (1:1000) were purchased from Cell Signaling Technology (Cambridge, MA, USA). MMP-3 (1:1000) was from Abcam Company (Cambridge, MA, USA). Goat anti-rabbit or anti-mouse IgG (1:10000 each; Jackson ImmunoResearch Laboratories).

### Small interfering RNA (siRNA) and transfection

For the RNA interfering experiment, SRC-siRNA: 5'-GCCTCAACGTGAAGCACTA-3', c-Jun-siRNA: 5'-TCCTGAAACAGAGCATGAC-3' and their scramble siRNA were purchased from Ribobio (Guangzhou, China). siRNA was transfected to 231/GEM cells at a final concentration of 100nM using Lipofectamine^™^ 2000 (Invitrogen, Carlsbad, CA, USA) according to the manufacturer’s protocol. Briefly, siRNA or scramble-siRNA and Lipofectamine 2000 were diluted in Opti-MEM medium respectively. Then, they were mixed at 1:1 ratio and incubated for 15 minutes. Finally, the mixture was added into FBS-free culture medium. After incubating with cells for 6 h, the FBS-free medium was replaced with complete medium.

The pENTER vector and pENTER/src-overexpressing human src plasmids were purchased from ViGene Biosciences (Shandong, China). MDA-MB-231 cells were transiently transfected by using the FuGENE^®^ HD Transfection Reagent (Promega), according to the manufacturer’s instructions. Briefly, 3μg plasmids and 9μl FuGENE transfection reagent were diluted in Opti-MEM medium respectively. Then, they were mixed at 1:1 ratio and incubated for 15 minutes. Finally, the mixture was added into the culture medium. 48 hours after transfection, the cells were collected for Western blot assay.

### Colony formation assays

In brief, 500 cells were seeded in 6-well plates in triplicate. On the next day, cells were treated with gemcitabine, saracatinib (AZD0530), and src-siRNA, either as a single agent or in combination. After 14 days of incubation, the colonies were fixed with 4% paraformaldehyde for 20 min and stained with 1% crystal violet for 30 min.

### Growth inhibition assays

Cells were seeded at a density of 8000 per well in 96-well culture plates and incubated overnight. The next day, gemcitabine or saracatinib (AZD0530) were added to each well at a gradient concentration and cells were incubated for another 48 h. Cell viability was assayed with a CCK-8 kit (Donjin Laboratories). Assessment of synergy effects was calculated by using Calcusyn computer program (Biosoft, Ferguson, MO). Values of combination index (CI) <1, = 1, >1 indicate synergism, additive effect, and antagonism, respectively [9].

### Cell migration assays

Migration experiments were carried out in chamber of 8μm transwell inserts (BD Falcon^™^; Becton Dickinson, Franklin Lakes, NJ, USA). An amount of 1×10^4^ 231/GEM cells were incubated in 200μL serum-free medium at the top chamber of each well insert, and 600μL 10% serum-containing medium were added to the lower chamber. Cells that migrated were fixed in 4% paraformaldehyde and stained with 1% crystal violet after 24 hours of incubation at 37°C. Stained cells were counted in five different fields in each well under an inverted microscope.

### Cell apoptosis analysis

The effects of various indicated treatments on cell viability were assessed using flow cytometry by staining with Annexin V/propidium iodide (PI) (BD Pharmingen). Briefly, cells were cultured for 48h after treatments and washed twice in ice-cold phosphate-buffered saline (PBS). A total of 2×10^5^ cells were resuspended in 100μL binding buffer, then 5μL Annexin V and 5μL PI was added. After 15 minutes of incubation in the dark, flow cytometry was performed.

### Western blot

Western blot analysis was performed according to the method described previously [10]. In brief, total protein lysates were obtained from cultured cells with a mixture of RIPA buffer (Beyotime, Shanghai, China) and protease inhibitor cocktail (Sigma) and PhosSTOP (Roche). Protein concentrations were determined using the BCA protein assay kit (Biyotime, Shanghai, China). Cell extracts (20μg/well) were electrophoresed on sodium dodecyl sulfate polyacrylamide Tris-HCl gels and transferred into polyvinylidene fluoride (PVDF) membranes. The membranes were then blocked with 10% skim milk in TBST for 1 h at room temperature and probed with the primary antibodies overnight at 4°C. After washing with TBST, the membranes were incubated with horseradish peroxidase (HRP)–conjugated secondary antibody for 1 h at room temperature, then washed three times with TBST, and detected by luminescent image analyzer (ImageQuant LAS4000 mini).

### Statistical analysis

Statistical analysis was performed using GraphPad Prism 5.0 software[11] (San Diego, CA). Differences between groups were calculated by using the Student’s *t*-test. The statistical significance was determined at *P*-value < 0.05.

## Result

### 231/GEM cell line showed elevated drug tolerance, enhanced proliferative and self-renewal abilities

**Fig 1:**
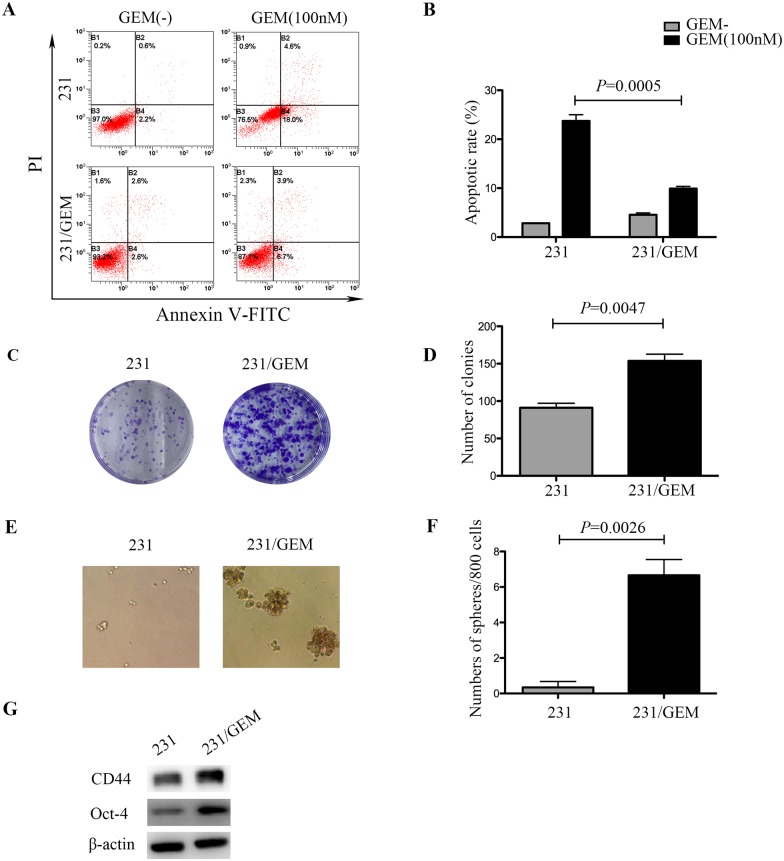
Characteristics of gemcitabine resistant breast cancer cells (231/GEM). (A) After exposed to 100nM GEM for 48h, drug-resistant cells exhibited stronger anti-apoptotic capacities than parental cells. (B) Quantitative analysis of apoptotic cells. Data are mean ± SEM and are representative of three independent experiments. (C-D) In comparison with the parental cells, drug-resistant cells got higher colony formation rate and the representative images were shown. Data are presented as mean ± SEM. (E-F) After cultured for 3 weeks using serum-free microsphere culture method, robust CSC self-renewal activity was observed in drug-resistant cells. The representative images were shown and data are mean ± SEM. (G) Western blot revealed that CSC associated markers CD44 and Oct-4 were elevated in drug-resistant cells.

To investigate the chemosensitivity of gemcitabine resistant breast cancer cell line, flow cytometry assay was conducted. After exposed to 100nM GEM for 48h, drug-resistant cells exhibited stronger anti-apoptotic capacities than the parental cells (Fig 1A and 1B). We also explored the distinction between 231/GEM and parental cells in proliferation ability and tumor stemness. In comparison with the parental cells, drug-resistant cells had higher colony formation rate (Fig 1C and 1D). Sphere forming assay was done using serum-free microsphere culture method for 3 weeks, and robust CSC self-renewal activity was observed in drug-resistant cells (Fig 1E and 1F). Further western blot analysis revealed that the expression of cancer stem cell (CSC) associated markers Oct-4 and CD44 were elevated in drug-resistant cells compared with parental cells (Fig 1G). Combined together, these data suggested that 231 cells gained enhanced drug tolerance, stronger proliferative and self-renewal abilities after acquirement of gemcitabine resistance.

### src inhibition had synergistic antitumor effects with gemcitabine and could partially reverse gemcitabine resistance

**Fig 2:**
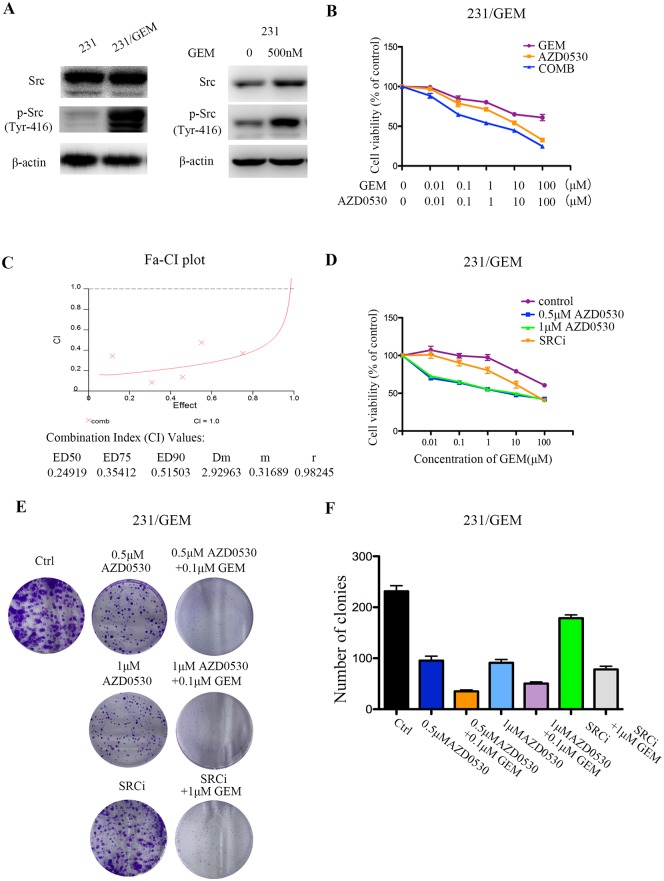
src inhibition partially reverses gemcitabine resistance in breast cancer cells. (A) Phosphorylation of src (Tyr416) was significantly elevated in gemcitabine resistant cells by western blot. src and phosphorylation of src (Tyr416) were also elevated in 231 cells after treated with 500nM gemcitabine for 48h. (B) Sensitivities of gemcitabine resistant cells to a series of concentrations of saracatinib (AZD0530), gemcitabine and their combination were assessed by CCK-8 assay (n = 5 per triplicate experiments). (C) Evaluation of synergism of gemcitabine and saracatinib (AZD0530) by isobologram analysis. Combination index (CI) value was analyzed by CalcuSyn software and plotted against Fa. (D) CCK-8 assay showed that src inhibition with AZD0530 and siRNA synergized with gemcitabine in drug-resistant cells (n = 5 per triplicate experiments). No difference between the concentrations of 500nM and 1μM AZD0530 was found. (E-F) Colony formation rate was detected after different treatments. Data are mean ± SEM and representative images were shown.

In this study, interestingly, over-activation of src kinase was found in gemcitabine resistant breast cancer cells. Western blot revealed that phosphorylation of src (Tyr-416) was significantly elevated in 231/GEM cells than in 231 cells. When treating the 231 cells with 500nM gemcitabine, the expression of src and p-src protein were increased, which further proved that gemcitabine stimulation could induce the over-expression of src protein (Fig 2A). Based on this, we hypothesis that whether inhibiting src kinase by siRNA or specific targeted drugs could reverse gemcitabine resistance in breast cancer. Then, saracatinib (AZD0530), a src inhibitor and src-siRNA were employed to assess the antitumor effects with gemcitabine in vitro by CCK-8 assay. First of all, sensitivities of gemcitabine resistant cells to a series of concentrations (0.01, 0.1, 1, 10, 100μM) of saracatinib (AZD0530), gemcitabine and their combination were assessed. Results showed the combination of AZD0530/gemcitabine dramatically reduced the viabilities of 231/GEM cells than single agent (Fig 2B). To better quantify the synergism effects, combination index (CI) value was analyzed by CalcuSyn software and plotted against Fa. The CI values at ED50, ED75, and ED90 were 0.25, 0.35 and 0.52, indicating AZD0530 could synergize with gemcitabine in 231/GEM cells (Fig 2C). Next, fixed concentrations of AZD0530 were evaluated. As shown in Fig 2D, cells survival was notably hampered when co-treated with gemcitabine and saracatinib (AZD0530), either at a concentration of 500nM or 1μM. In addition, the knockdown of src by siRNA had some level of synergistic effect with gemcitabine, which was less prominent than saracatinib (AZD0530) (Fig 2D). The colony formation assays displayed a suppressed proliferative ability after independent treatment of saracatinib (AZD0530) or src-siRNA, and the colony formation rate was remarkably reduced after combination of src inhibition and gemcitabine (Fig 2E and 2F).

### src inhibition enhances gemcitabine induced apoptosis in 231/GEM cell line

**Fig 3:**
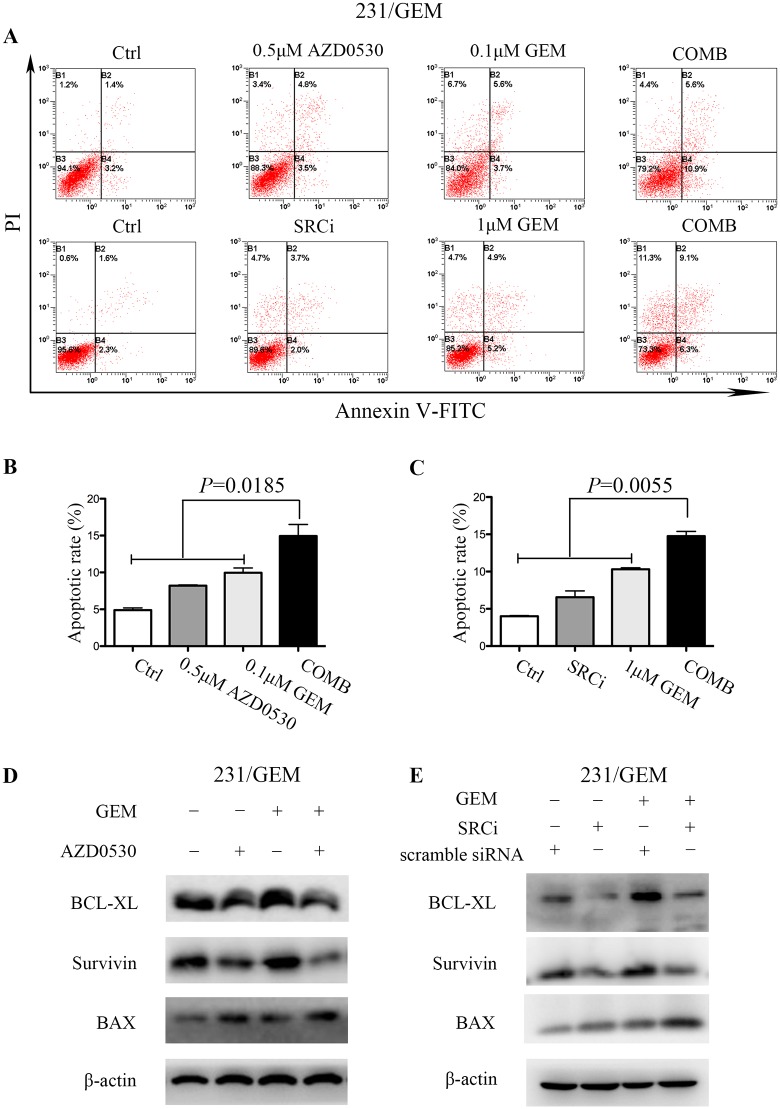
src inhibition enhances gemcitabine induced apoptosis in drug-resistant cells. (A) The apoptosis induced by src inhibition and gemcitabine was detected by flow cytometry. After treated with 500nM AZD0530, 100nM GEM and their combination for 48h, the Comb group exhibited increased apoptosis than treated alone. Similarly, src inhibition with siRNA combined with 1μM GEM showed increased apoptosis than treated alone. (B-C) Quantitative analysis of apoptotic cells. Data are mean ± SEM and are representative of three independent experiments. (D-E) The expression levels of pro-apoptotic and anti-apoptotic associated proteins were detected by western blot.

The combination of saracatinib/src-siRNA and gemcitabine was also investigated on their induction of apoptosis. After treated with 500nM saracatinib (AZD0530), 100nM GEM and their combination for 48h, the combination group exhibited increased apoptosis than treated alone (Fig 3A and 3B). It is noteworthy that src inhibition with siRNA alone did not promote apoptosis, which was differed from saracatinib (AZD0530). Also, only when 1μM GEM, rather than 100nM GEM was combined with siRNA did we observed an increased apoptosis (Fig 3A–3C). Western blot analysis was performed to detect apoptotic cell death with molecular biomarkers of apoptosis, as expected, the combination of src inhibition and gemcitabine significantly increased the expression of pro-apoptotic protein BAX, and decreased the level of anti-apoptotic proteins BCL-XL and Survivin (Fig 3D and 3E). Taken together, these results suggest that treatment of saracatinib/src-siRNA significantly enhances gemcitabine-induced apoptosis in 231/GEM cell line.

### src inhibition attenuates gemcitabine resistance associated migration and stem cell capacities

**Fig 4:**
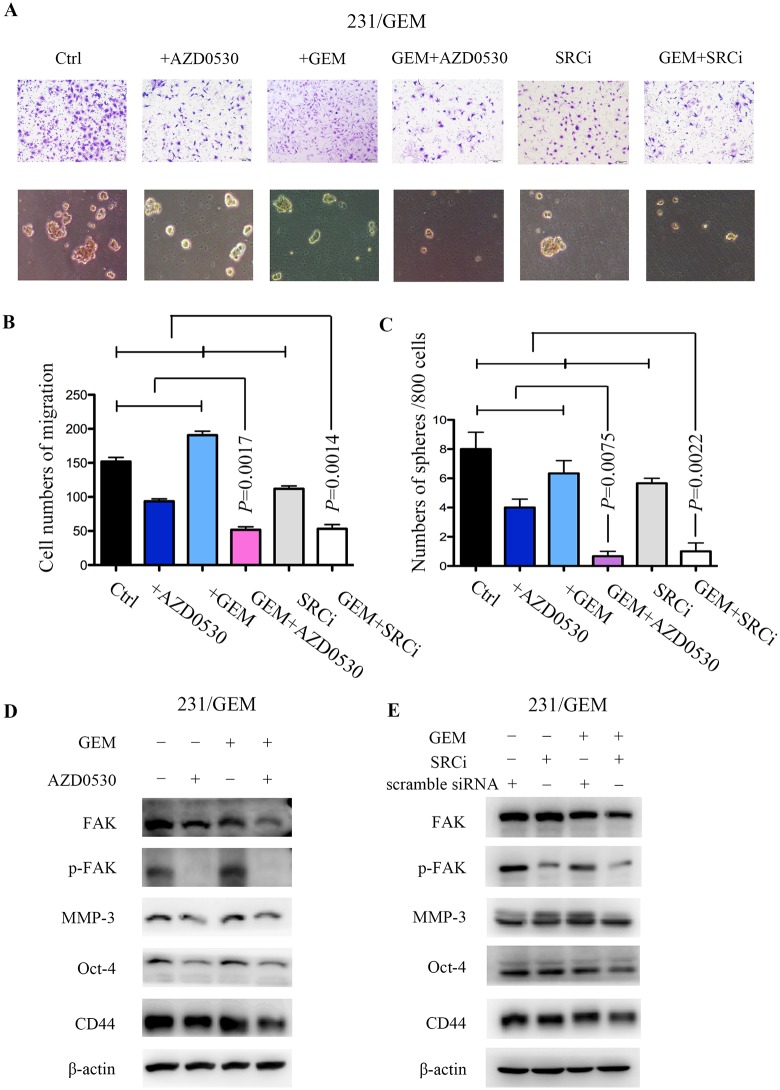
src inhibition attenuates gemcitabine resistance associated migration and stem cell capacities. (A, C) Transwell assay was utilized to evaluate the migration abilities of drug-resistant cells after indicated treatments (n = 3 per triplicate experiments), bars 100μM. Data are means ± SEM. (B, D) After indicated treatments, drug-resistant cells were cultured in serum-free microsphere for 3 weeks and CSC self-renewal activity was assessed (n = 3 per triplicate experiments). Data are means ± SEM. (E-F) Migration and stem cell associated proteins were evaluated in drug-resistant cells by western blot.

Transwell assay was utilized to evaluate the variance of migration ability after distinct treatments in 231/GEM cell line. Here, we found the saracatinib (AZD0530) or src-siRNA alone could inhibit gemcitabine resistance associated migration, whereas gemcitabine did not exert any effect. The cooperation of src inhibition and gemcitabine significantly retarded the migration of 231/GEM than monotherapy (Fig 4A and 4B), along with subdued expression of migration associated proteins p-FAK and MMP-3 (Fig 4D and 4E).

In addition, after indicated treatments, drug-resistant cells were cultured in serum-free microsphere for 3 weeks and CSC self-renewal activity was assessed. Treatment with low doses of saracatinib/gemcitabine inhibited sphere forming, and the combinatorial action of src inhibition and gemcitabine dramatically weakened the efficiency of sphere forming compared with either agent alone (Fig 4A–4C). Accordingly, the remarkable decrease of cancer stem cell markers, CD44 and Oct-4, also reflected the synergistic inhibition of cancer stemness (Fig 4D and 4E).

### src inhibition cooperates with gemcitabine partially through the AKT/c-Jun pathway

**Fig 5:**
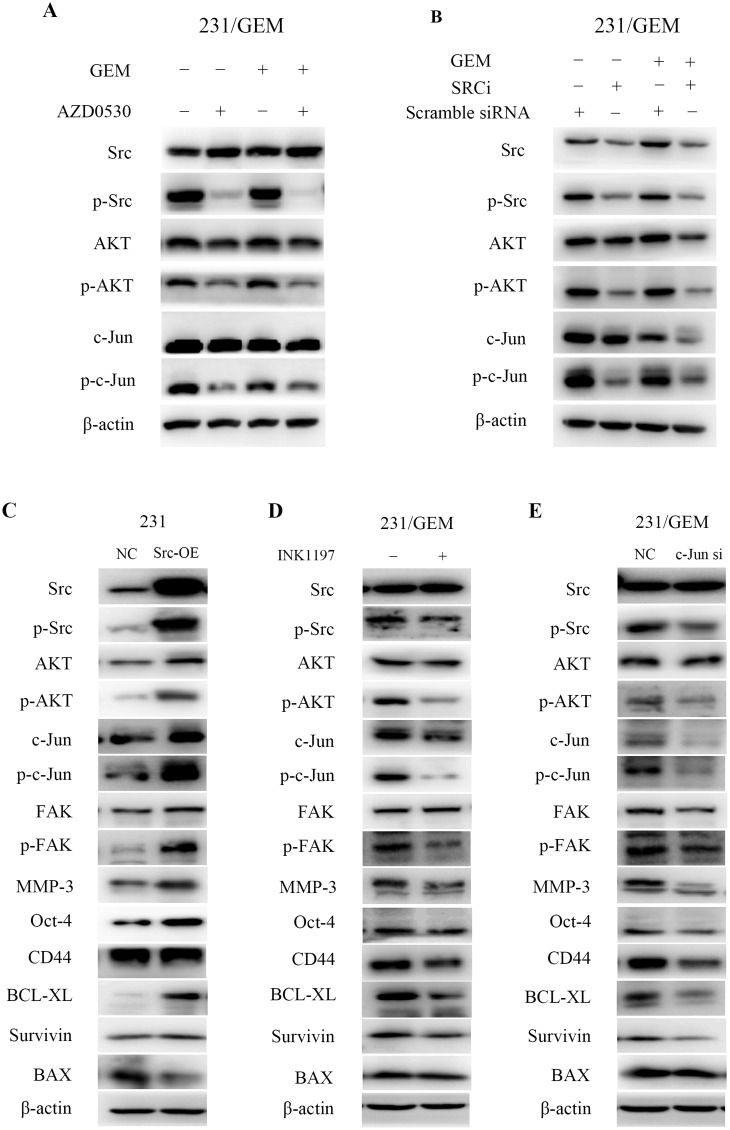
src inhibition cooperates with gemcitabine through the AKT/c-Jun pathway. (A) The variation of src/p-Src, AKT/p-AKT, c-Jun/p-c-Jun protein level after treated with AZD0530, gemcitabine or both. (B) The variation of src/p-Src, AKT/p-AKT, c-Jun/p-c-Jun protein level after treated with src-siRNA, scramble siRNA, gemcitabine or both. (C) The variation of signaling pathway-related and effector proteins in src overexpressed-231 cells. (D-E) The variation of signaling pathway-related and effector proteins after PI3K selective inhibitor INK1197 treatment and c-Jun siRNA transfection.

To further elucidate the underlying mechanisms, we explored the potential signaling pathways involved in the joint action of src inhibition and gemcitabine. The western blot analysis showed that saracatinib (AZD0530) treatment or src-siRNA decreased the expression of p-Src (Tyr416). When phosphorylation of src was inhibited, the expression of p-AKT was simultaneously down-regulated with a concomitant reduce in p-c-Jun. These phenomena were more pronounced when combined with gemcitabine treatment (Fig 5A and 5B). To further verify the AKT/c-Jun signaling pathway, we analyzed the effects of src, AKT and c-jun expression changes on the downstream effector proteins. The results showed that src overexpression in 231 cells increased src/p-Src, AKT/p-AKT, c-Jun/p-c-Jun protein levels and enhanced the expression of effector proteins like FAK/p-FAK, MMP3, Oct-4, BCL-XL, Survivin with a decreased expression of BAX (Fig 5C). no significant difference of CD44 was observed. In addition, both PI3K selective inhibitor INK1197 treatment and c-Jun siRNA transfection in 231/GEM cells significantly decreased p-Src, p-AKT, c-Jun and p-c-Jun, along with a suppressed p-FAK, MMP3, Oct-4, CD44, BCL-XL and Survivin expression, but not an elevated BAX expression (Fig 5D and 5E) was observed. These data indicated that src inhibition cooperated with gemcitabine may partially through the AKT/c-Jun pathway.

## Discussion

Gemcitabine-based chemotherapy remains the standard approach in metastatic breast cancer [12, 13]. However, the occurrence of gemcitabine resistance inevitably results in chemotherapeutic failure and relapsed tumor. We initially identified the aberrant activation of p-Src in gemcitabine-resistant triple-negative breast cancer (TNBC) cells and then explored the potential of src inhibition as a feasible treatment strategy. As results, we found that the inhibition of src by saracatinib (AZD0530) or siRNA partially reversed gemcitabine resistance in 231/GEM cells and attenuated gemcitabine resistance associated anti-apoptosis, migration and stem cell capacities. Further western blot analysis revealed that the combination up-regulated pro-apoptotic protein BAX, along with the down-regulation of anti-apoptotic proteins (BCL-XL, Survivin), migration associated proteins (p-FAK, MMP-3) and CSC markers (CD44, Oct-4), which was probably mediated by AKT/c-Jun pathway.

Overexpression of src is associated with malignant cellular behavior[5, 6], therefore, inhibition of src appears to be a rational therapeutic strategy. Several clinical studies have evaluated the use of selective src inhibitors like Dasatinib or Saracatinib for treating breast cancer [14, 15]. Preclinical data demonstrated saracatinib decreased cell growth and migration in MDA-MB-231 breast cancer cell line and xenograft models, however, it was later shown to be ineffective as single agent therapy in clinical trials [16, 17]. Actually, oncogenic activation of various growth factor pathways can lead to chemo-resistance, hence, monotherapy with signal transduction inhibitor may have only modest antitumor efficacy. Previous studies have shown that src inhibition may heighten gemcitabine cytotoxicity in pancreatic adenocarcinoma cells, indicating combined chemo/signal transduction inhibitor therapy may cause greater growth inhibition and delay emergence of resistance [18, 19]. Our results also confirmed that when src inhibition and gemcitabine were combined, anti-tumor effects (proliferation, migration, apoptosis and stemness) were maximized compared with either agent alone in gemcitabine-resistant triple-negative breast cancer cells.

Noticeably, in comparison with saracatinib (AZD0530), src-siRNA did not alter the expression of BAX, Oct-4 and CD44 in our study. This may due to the different functional mechanisms between molecular inhibitors and siRNAs. Inhibitors mainly target protein level directly and may affect more than one kind of proteins. For example, saracatinib (AZD0530) can not only inhibit c-src, but also inhibit c-Yes, Fyn, Lyn, Blk, Fgr and Lck [7]. However, siRNA specifically influence mRNA expression of a certain gene, thereby affecting a single protein. Hence, saracatinib (AZD0530) may affect the expression of some proteins but siRNA may not. Another possible reason may be the cytotoxicity effect of saracatinib (AZD0530), which can cause the changes of apoptosis-, migration- and stemness- associated proteins. As for src-siRNA, itself may not affect all the above proteins, but when combined with GEM, they exert synergistic effect and thus alter the expressions of these proteins.

Remarkably, we for the first time confirmed the enhanced self-renewal abilities in 231/GEM cell line and demonstrated that src inhibition will hamper stemness of 231/GEM by suppressing sphere formation. CSCs, a subpopulation within the tumor mass that can self-renew and maintain the tumor presence, are characterized by the cause of disease relapse and chemo-resistance [20–22]. Commonly used anti-cancer drugs are not capable of eradicating CSCs due to their robust survival mechanisms. src has been substantiated owning pleiotropic functional activity, including supporting CSC phenotype in some cancer types, but reports of its involvement in breast cancer are limited [23]. Herein, we revealed the combinatorial action of src inhibition and gemcitabine dramatically weakened the efficiency of sphere forming compared with either agent alone, highlighting a possibility of using src inhibition with chemotherapeutic drugs to subdue CSCs proliferation and self-renewal.

Breast cancer cells depend heavily on the AKT pathway as a survival factor or "molecular crutch" [24, 25]. Over-activation of AKT signaling contributes to eluding the effects of cytotoxic agents and sustaining cell viability [26, 27]. Our western blot analysis showed that saracatinib caused a decrease in AKT (Ser473) phosphorylation and it might be one of the mechanism to reverse resistance in breast cancer. c-Jun is a component of the transcription factor activator protein 1 (AP-1), which participates in the diverse tumor end points like proliferation, transformation, differentiation, and apoptosis [28–30]. Prior studies have substantiated the functional link between activated c-Jun and tumorigenesis or metastasis in breast cancer both *in vitro* and *vivo* [31, 32]. Our results indicated that along with the restraint of p-Src and p-AKT, the phosphorylation of c-Jun was inhibited, particularly under the joint effects of src inhibition and gemcitabine, which is in line with their inhibitory effects on deterring carcinogenesis and cancer progression.

In summary, these data suggest that integration of src inhibition with gemcitabine therapy may present a useful treatment regimen to overcome gemcitabine resistance and to inhibit tumor growth, metastasis and recurrence of triple-negative breast cancer cells. AKT/c-Jun pathway may be a therapeutic target in gemcitabine-resistant breast cancer patients in clinic. Further prospective validation in xenograft models or a large set of patients is required to corroborate our results.

## Abbreviations

CSC: cancer stem cell
GEM: gemcitabine
TNBC: triple-negative breast cancer
231/GEM: MDA-MB-231 gemcitabine resistant cell line
231: MDA-MB-231
